# Novel water filtration of saline water in the outermost layer of mangrove roots

**DOI:** 10.1038/srep20426

**Published:** 2016-02-05

**Authors:** Kiwoong Kim, Eunseok Seo, Suk-Kyu Chang, Tae Jung Park, Sang Joon Lee

**Affiliations:** 1 Department of Mechanical Engineering, Pohang University of Science and Technology (POSTECH), San 31, Hyoja-dong, Pohang 790-784, Republic of Korea; 2 Department of New Biology, Daegu Gyeongbuk Institute of Science and Technology (DGIST), 333, Techno Jungang-daero, Daegu, 711-873, Republic of Korea; 3 Department of Chemistry, Chung-Ang University, 84 Heukseok-ro, Dongjak-gu, Seoul 06974, Republic of Korea

## Abstract

The scarcity of fresh water is a global challenge faced at present. Several desalination methods have been suggested to secure fresh water from sea water. However, conventional methods suffer from technical limitations, such as high power consumption, expensive operating costs, and limited system durability. In this study, we examined the feasibility of using halophytes as a novel technology of desalinating high-concentration saline water for long periods. This study investigated the biophysical characteristics of sea water filtration in the roots of the mangrove *Rhizophora stylosa* from a plant hydrodynamic point of view. *R. stylosa* can grow even in saline water, and the salt level in its roots is regulated within a certain threshold value through filtration. The root possesses a hierarchical, triple layered pore structure in the epidermis, and most Na^+^ ions are filtered at the first sublayer of the outermost layer. The high blockage of Na^+^ ions is attributed to the high surface zeta potential of the first layer. The second layer, which is composed of macroporous structures, also facilitates Na^+^ ion filtration. This study provides insights into the mechanism underlying water filtration through halophyte roots and serves as a basis for the development of a novel bio-inspired desalination method.

The lack of potable water has become a serious global issue for a long time^1^. A promising method to resolve this water crisis is the desalination of seawater, which accounts for 97% of all available water resources on earth^2^. Distillation and reverse osmosis are the conventional desalination methods used at present; however, these methods display several problems, including high energy consumption^3^, treatment of membrane fouling, and short durability^4^. Thus, a new concept for the effective desalination of seawater is inevitably required to resolve this water crisis.

Uptake of Na^+^ ions is desirable for halophytes to build up osmotic potential, absorb water and sustain turgor pressure. However, excess Na^+^ ions may work on toxic element. Therefore, halophytes try to adjust salinity delicately between growth and survival strategies. In this point of view, a novel sustainable desalination method can be derived from halophytes, which are in contact with saline water through their roots. Halophytes exclude salt through their roots, secrete the accumulated salt through their aerial parts, and sequester salt in senescent leaves and/or the bark^5^,^6^,^7^. Mangroves are facultative halophytes, and *Bruguiera* is known for its special ultrafiltration system that can filter approximately 90% of Na^+^ ions from the surrounding seawater through the roots^8^,^9^,^10^. The species also exhibits a high rate of salt rejection. The water-filtering process in mangrove roots has received considerable attention for several decades^11^,^12^,^13^. However, the detailed ultrafiltration mechanisms remain unclear yet. Morphological structures of plants and their functions have been evolved through a long history to survive against harsh environmental conditions^14^. In this aspect, mangrove roots may enact a novel ability of long-lasting desalination of saline water with a high salt rejection rate.

This study investigated the filtration of salt from saline water through the roots of *Rhizophora stylosa* (RS), a species of mangroves (*Bruguiera*), from a biophysical point of view. We identified the special morphological structures of RS for regulating salinity in surrounding seawater. We also conjectured a plausible seawater filtration mechanism of halophyte roots on the basis of the revealed morphological structures of the roots and the electrokinetic characteristics used for the sustainable and efficient filtration of saline water. The results of this study may serve as a basis for the development of innovative bio-inspired desalination devices.

## Results

### Filtering characteristics of RS roots

**Figure 1:**
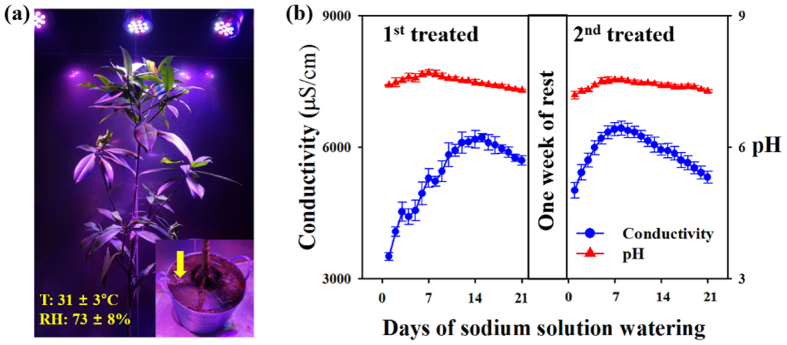
Filtration of Na^+^ ions from surrounding saline solution by *Rhizophora Stylosa*. (**a**) *Rhizophora Stylosa* (RS) was used as a test sample. Conductivity and pH were measured around RS roots. The yellow arrow indicates the measurement point. (**b**) Temporal variations in the conductivity and pH of saline solution, showing the filtration characteristics of Na^+^ ions. Conductivity increased up to approximately 6100 μS/cm and eventually decreased, whereas the pH remained in the range of 7.2–7.7. Error bar indicates standard deviation (*n* = 10).

Temporal variations in the conductivity and pH of saline solution surrounding RS roots were monitored for 6 weeks to examine the performance of sodium filtration. A 3.5 wt.% sodium solution was used to water the first sodium-treated pot every day for 3 weeks and again for 3 weeks to the same pot (the second sodium-treated pot) after a one-week break. As shown in Fig. 1b, the pH in the first sodium-treated pot slightly increased from 7.4 to 7.7 for 7 days and then decreased to 7.3 during the succeeding 2 weeks. The pH in the second sodium-treated pot similarly increased from 7.2 to 7.5 for 8 days and then decreased to 7.3 during the next 13 days. The conductivity measured in the pot indicated sodium concentration and showed large temporal variations. The conductivity in the first sodium-treated pot increased within the initial 15 days from 3507 μS/cm to 6220 μS/cm and then decreased to 5701 μS/cm. However, similar to that in the first pot, the conductivity in the second sodium-treated pot increased within the initial 8 days and then decreased to 5315 μS/cm. On average, the conductivity increased up to 6220 μS/cm (first sodium-treated pot) and 6335 μS/cm (second sodium-treated pot), and then decreased with time (Table 1). However, the pH in the pot was almost maintained at virtually constant levels to minimize the effect of pH on conductivity variation because of sensitivity to slight changes in pH^15^. During a one-week break, pH and conductivity decreased more quickly.

The conductivity of the surrounding saline solution increased up to approximately 6100 μS/cm and then decreased, whereas the pH remained in the range of 7.2 to 7.7. Regardless of the initial conductivity, the conductivity of saline solution increased up to 6100 μS/cm and then decreased. The conductivity in the first sodium-treated pot was increased, because Na^+^ and Cl^−^ ions were also augmented in the pot. Variation of conductivity according to concentration of sodium solution wt.% is depicted in Fig. S1. According to this trend, the conductivity in the pot should be continuously increased. However, mangroves adjust the salinity of the surrounding environment, when the concentration of sodium solution in the pot becomes too high. Therefore, sodium ions in the pot are absorbed into the roots of RS and filtered. This response may be one of the survival strategies of mangroves because copious Na^+^ ions are extremely harmful even to halophytes.

### Morphological features of RS roots

**Figure 2:**
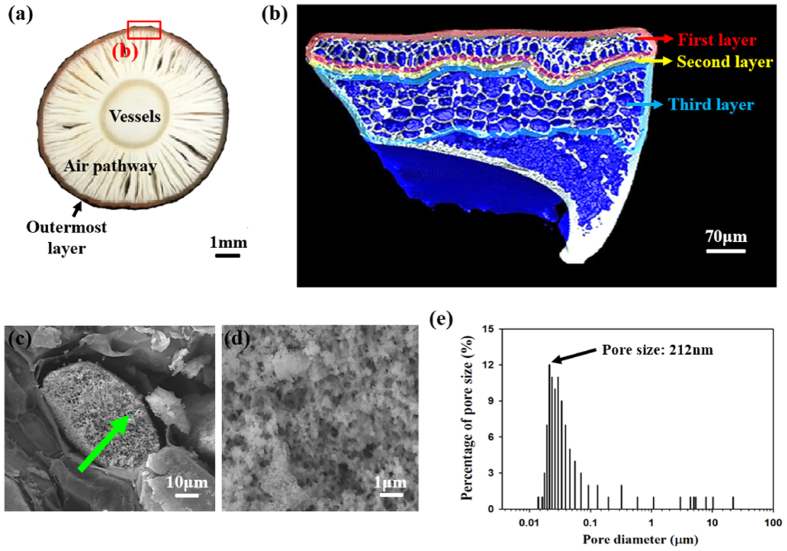
Triple-layered porous structure of RS roots. (**a**) Cross-sectional image of a mangrove root, exhibiting the outermost layer, air pathway, and vessels. (**b**) Reconstructed X-ray image shows the internal morphological structure of the outermost root layer. The outermost layer consists of three parts with different morphologies and functions marked as red region (first layer), yellow region (second layer), and blue region (third layer). (**c**) The second layer contains a porous membrane, whose pore size is in a scale of hundreds of nanometers. (**d**) Magnified view of the membrane, indicated by a green arrow in (**c**). Nanometer scale pores are irregularly distributed with various structures like conventional membrane filter. (**e**) Percentage distribution of pore-size measured by a mercury porosimetry. Pores of the membrane in the second layer are mainly at hundreds of nanometers in size. Size of 212 nm pores indicated by black arrow are the most.

The morphological structures of RS roots were visualized using various imaging techniques. Figure 2a shows a typical cross-sectional image of the root. The cross section of the root is primarily divided into three distinct parts: outermost layer, air pathway, and vessels. The air pathways in the root can similarly be found in other aquatic plants living in muddy areas^16^. Procuring air through the root is difficult without such air pathways. The root consists of epidermis, endodermis, parenchyma, xylem, and phloem vessels^17^. The constituent elements of RS roots are similar to those of glycophytes. However, the outermost layer of the root shows different characteristics^18^. The outermost layer of RS can be divided into three parts as shown in Fig. 2b to discriminate their anatomy and functions. Interestingly, the second layer contains macroporous membranes whose diameters are in a scale of hundreds of nanometers (Fig. 2c). Figure 2d is a magnified image of the membrane indicated by an arrow in Fig. 2c. In the membrane, pores are irregularly distributed with different pore sizes. As shown in Fig. 2e, the percentage of pore size show normal distribution. The pores of 212 nm size are mainly existed and average diameter of pore size is 324 nm. The morphological structures of other parts in the outermost layer are shown in Fig. S5.

### Filtration of Na^+^ ions in the roots

Among various biophysical phenomena in mangrove roots, air gap is assumed to be used for filtering solutes. When negative pressure is applied to nanoporous structures with hydrophobic surfaces, solutes are rejected from passing through the surface, whereas only water is filtered through evaporation, transmission, and condensation processes^19^. Another mechanism of filtering Na^+^ ions is a physical barrier that blocks the apoplastic bypass passage of ions^20^. However, this physical barrier faces a fouling problem induced by continuous water filtration. Among various physical phenomena, the charge effect is one of the most important factors. The electrostatic interaction of charged ions leads to ion accumulation near the membrane surface or passage through porous structures^21^. This accumulation engenders the gathering of counter ions with a sign opposite to that of the surface charge of the membrane. To verify this hypothesis, we visualized the accumulation and translocation of Na^+^ ions by using a Na^+^-specific fluorescent indicator (sodium tetramethylammonium; STTMA) and measured the surface ζ-potential of the roots.

**Figure 3:**
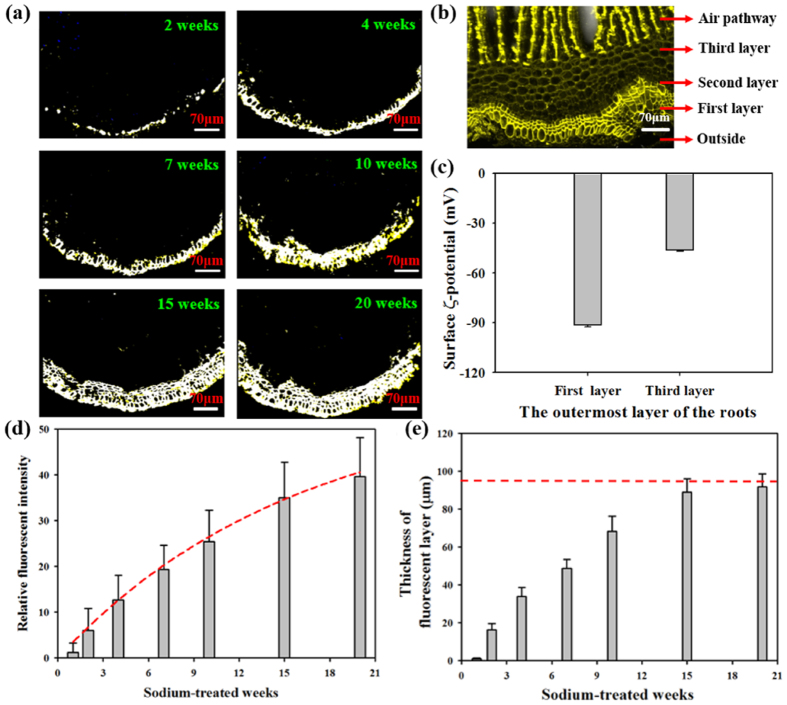
Accumulation of Na^+^ ions in RS roots. (**a**) Visualization of Na^+^ ions using a Na^+^ specific fluorescent dye (STTMA). Fluorescent images were acquired by a two-photon microscopy. As the watering period was prolonged, fluorescence became clearly detectable at the first sublayer of the outermost layer. (**b**) Confocal micrograph of the outermost layer of the root. First layer and air pathway show relatively strong auto-fluorescence. Each layer is obviously observed. (**c**) Surface ζ-potential of the first and third layers. The first layer had large ζ-potential value, −91.4 ± 0.93 mV. The ζ-potential of third layer was −46.2 ± 0.78 mV. (**d**) Temporal variation in relative fluorescent intensity obtained by averaging 15 images. As marked by a red dashed line, the relative fluorescent intensity steadily increased with prolonged sodium treatment. (**e**) The thickness of the fluorescent layer increased up to the thickness of the first sublayer of the outermost layer, as marked by a red dashed line. Error bars in (**c–e**) indicate standard deviations (*n* = 3) and (*n* = 15).

Figure 3a shows the increased accumulation of Na^+^ ions with time. These fluorescence images indicate that free Na^+^ ions are mostly localized around the outermost layer. Fluorescence became slightly detectable after 2 weeks of watering with the sodium solution. Fluorescence was clearly detected when the watering period was prolonged. After 4 weeks, fluorescence was mainly perceived along with the outer part of the first layer. After 15 weeks of watering with the sodium solution, the first layer of the roots became totally visible (Fig. 3a,b). This results suggests that the first layer, which is the outermost layer of the roots, mainly entraps intracellular free Na^+^ ions. In addition, Na^+^ ions cannot penetrate into the second layer of the roots even with 20 weeks continued supply of sodium solution. The hydrostatic pressure of xylem sap is estimated to be at least −24 atmospheres^22^. This would balance with osmotic potential of sea water with allowing turgor in the cells. Although the hydraulic drag exerting on the root surface is relatively high, Na^+^ ions are mostly filtered out at the first layer without penetrating into the second layer of the roots during 20 weeks continued supply of the sodium solutions.

The surface ζ-potential of the first layer was approximately −91.4 ± 0.93 mV, which is considerably larger than those of conventional filtration membranes^21^ and the third layer showed −46.2 ± 0.78 mV (Fig. 3c). Particle separation is commonly affected by the steric effect because of the small size of pores^23^. The filtration of ionic solutes by membranes relies on the charge effect, which performs an important function in the purification performance of porous membranes. Given its negative ζ-potential, the membrane surface possesses ion selectivity. The concentration of counter ions with an opposite sign of charge to the surface ζ-potential of the membrane is higher on the surface of the membrane than in the bulk solution. However, the concentration of the co-ions with the same charge as the surface charge of the membrane is smaller on the membrane surface^24^. A potential difference at the interface, termed the Donnan potential, was created to counteract the transport of counter ions into the roots of RS. Therefore, Na^+^ ions accumulated on the first sublayer of the outermost layer.

As shown in Fig. 3d,e, the relative fluorescent intensity gradually increased with prolonged sodium treatment. In addition, the fluorescent layer thickness increased to the thickness of the first sublayer of the outermost layer (97 μm), as marked by a dashed line. In the glycophytes, Na^+^ ions from the sodium solution used for watering were absorbed by the entire roots^25^. However, Na^+^ ions were only absorbed on the first sublayer of the outermost layer in the RS roots.

### Feasibility test of water filtration under *in vitro* conditions

**Figure 4:**
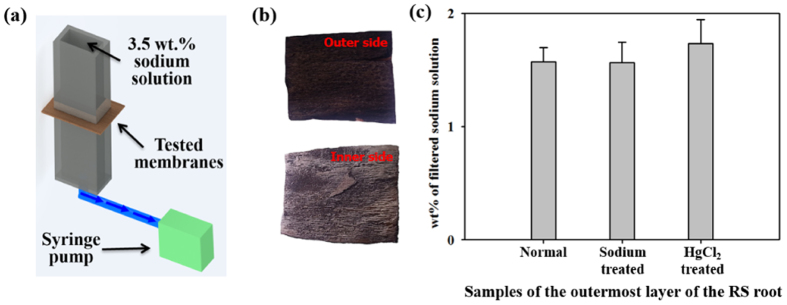
Water filtration experiment under *in vitro* conditions. (**a**) Schematic of the experimental setup. Three types of prepared membranes: normal root without pretreatment, sodium-treated root treated by sodium solution for 4 weeks, and HgCl_2_-treated root whose aquaporins were blocked. Each of the three sliced outermost layers was attached at the middle section of a square channel, and a 3.5 wt.% sodium solution (9700 μS/cm) was supplied from the top of the channel. Suction pressure was applied by a syringe pump. (**b**) In the experiment model, the outer side of the sliced outermost layer was headed to upper direction and the inner side was faced to the bottom direction. The outer side of the root contacted the sodium solution directly like actual root. (**c**) wt.% of filtered sodium solution of the three root models. The filtered sodium solutions of the normal and sodium treated roots presented similar average values of 1.56 and 1.57 wt.%, respectively. The wt.% of the sodium solution filtered by the HgCl_2_-treated root presented a slightly larger value.

Mangroves filter Na^+^ ions effectively by using the outermost root layer. Thus, the internal structures of mangroves can absorb relatively pure water by filtering the Na^+^ ions of seawater on the roots. The desalination rate of the outermost layer was investigated to confirm the feasibility of RS roots for biomimicry. Figure 4a shows a schematic of the experimental setup. The sliced outermost layer of the roots was attached as a test membrane. A 3.5 wt.% sodium solution (9687 μS/cm) was supplied while applying suction pressure using a syringe pump. At the start of the infiltration process, the initial pressure at the tested membrane was 101.31 kPa. As shown in Fig. S6b, the applied pressure was rapidly decreased to 22.39 kPa for the initial 70 sec. Thereafter, it was asymptotically decreased from 22.39 kPa to 3.61 kPa.

As shown in Fig. 4c, 62% of Na^+^ ions were filtered on the average when normal roots were installed as a membrane. Meanwhile, the filtered sodium solutions presented an average value of 1.57 wt.% (4169 ± 107 μS/cm) when the sodium-treated roots whose outside of the outermost layer was covered with thin Na^+^ ions (Fig. 3a) were applied. This value is similar to that of normal roots (1.56 wt.%, 4152 ± 126 μS/cm). In addition, water flux of both case is also almost same. These results imply that the filtration rate of the outermost layer is not sensitive to sodium treatment and that the filtration function of RS remains intact under this treatment. Therefore, RS roots can continuously filter Na^+^ ions without a significant fouling problem. When HgCl_2_-treated roots with blocked aquaporins were used as a membrane, the filtered sodium solutions presented an average value of 1.73 wt.%, which is larger than the previous two values. Water flux passing through the *in-vitro* model can be estimated using the equation of 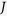 = *Q*_*p*_/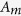, where 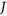 is the flux (L/h/m^2^), *Q*_*p*_ is the filtration rate through the membrane (L/h), and 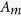 is the surface area of the membrane (m^2^). The estimated water flux of the normal and sodium treated roots is about 11 L/h/m^2^ and that of the HgCl_2_-treated root is around 7 L/h/m^2^. The presence of aquaporins in membranes increases water permeability along with the cell-to-cell pathways^26^. These experimental results indicate that aquaporins help promote root hydraulic conductivity without considerably influencing the filtration rate.

## Discussion

The design of a water purification device can be bioinspired by halophytes as a good candidate for sea water desalination. However, the hydrodynamic advantages of halophytes have not been completely understood because the underlying biophysical features of the water filtration mechanisms of RS remain unclarified. In this study, the morphological, functional, and chemical properties of RS roots were analyzed and a feasibility test of water filtration under *in vitro* conditions was conducted to understand the effective water filtration.

**Figure 5:**
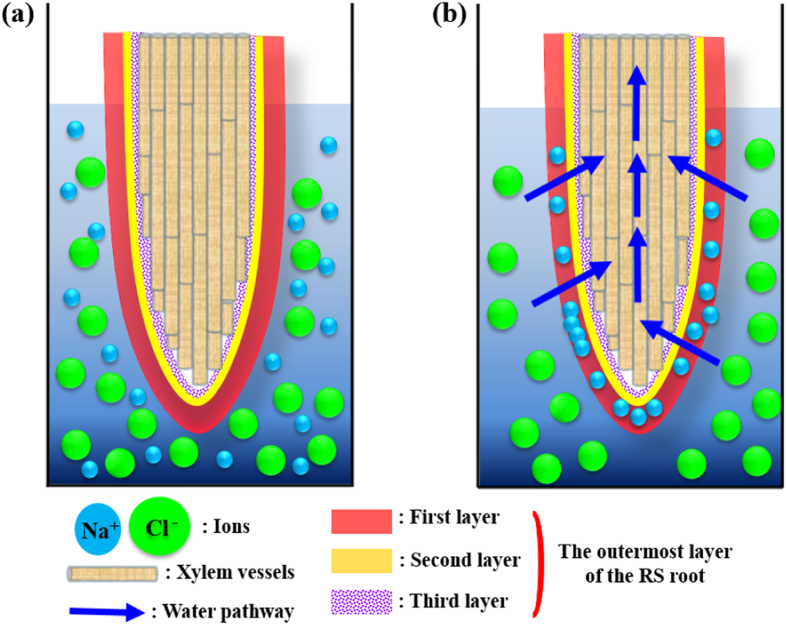
Schematic of water filtration in RS roots. (**a**) Schematic of an RS root. The outermost layer is composed of three layers. The root is immersed in NaCl solution. (**b**) Water pass through the outermost layer when a negative suction pressure is applied across the outermost layer. The Donnan potential effect repels Cl^−^ ions from the first sublayer of the outermost layer. Na^+^ ions attach to the first layer to satisfy the electro-neutrality requirement, and salt retention eventually occurs.

As shown in Fig. 5, water pass through the outermost layer when a hydraulic pressure gradient is applied across the outermost root layer. The Donnan potential repels Cl^−^ ions from the first sublayer of the outermost layer, which possesses a relatively large surface ζ-potential. Na^+^ ions attach to the first layer to satisfy the electro-neutrality requirement, and salt retention eventually occurs^27^. Na^+^ ions are filtered on mangrove roots through the surface charge effect. The rejection of cations is enhanced when the zeta potential of a membrane is positive. However, if positively-charged membranes were employed in the present study, multivalent cations would be removed from water. Divalent and multivalent cations are rejected by the strong electrostatic repulsion between higher valence cations and positively-charged membranes, while the rejection to monovalent ions is lower, e.g. NaCl(47.6%)^28^. The second layer, which bears tens of nanometer-scale porous structures, also helps protect Na^+^ ions from entering the xylem vessels of the roots. In addition, muddy environments, which are characterized by a low Peclet number and a short characteristic length, are the usual habitat of mangroves. Muddy environments also facilitate the effective purification of saline water.

However, fouling remains unresolved in conventional desalination treatment. As shown in Fig. 3a, Na^+^ ions were almost completely accumulated in the first sublayer of the outermost layer. The accumulated ions in the first layer may induce very high osmotic pressure. This increased osmotic pressure may enhance the filtration process. The hydraulic resistance in the roots of plant is known to be much larger than other parts of the plants^29^. In this point of view, high osmotic pressure of the accumulated sodium ions in the first layer would be helpful for water absorption from outside into the roots. Due to technical limitations of currently available advanced imaging techniques, *in vivo* observation of real water-filtration process in the roots of RS is nearly impossible. In addition, the *in-vitro* model tested in this study is not suitable for checking this infiltration phenomenon. As a succeeding research, the fouling problem in mangroves needs to be revealed by fabricating *in vitro* experimental models based on the morphological features of mangrove roots. Although Na^+^/H^+^ antiporters were not handled in this study, their association with sodium association needs to be considered in future research.

The present results are not only useful in understanding the underlying water filtration mechanism of halophytes but also in providing the database required for developing practical engineering applications. This study may also be used as a basis to develop a new innovative biomimetic desalination device. This type of biomimetic desalination technology would be ultimately beneficial for resolving the serious water shortage problems that we will face in the near future.

## Methods

### Plant sample

Well-grown and healthy *RS* plants (Mirim Development Co., Jeju, Korea) were used in this experiment. Water absorption of plants are closely related with evaporation and transpiration at their leaves^30^. The evaporation and transpiration are important factors to water absorption at the roots of plants. Test samples were cultivated in an air-conditioned chamber in which temperature and humidity were constantly maintained (Fig. 1a). During the experiment, the temperature and relative humidity of the chamber were maintained at 31 ± 3 °C and 73 ± 8%, respectively. LED lamps (PGL-E15, PARUS, Cheonan, Korea) were used to irradiate 430 nm:460 nm:660 nm light. A 3.5 wt.% sodium solution was watered in a pot to check temporal variations. Except on the first day, 0.5 L of sodium solution was watered every day for 3 weeks into the pot, which was called the first sodium-treated pot. After a one-week break, the sodium solution was again used to water the same pot. This pot was then called the second sodium-treated pot. Conductivity and pH were measured at several points in the pot (Fig. 1b). Measurements were repeated for 10 times daily and by using a conductivity and pH probe connected to a Vernier LabQuest (Veaverton, OR, USA).

### Sample preparation for synchrotron X-ray micro CT

All materials used for contrast staining and sample preparation for synchrotron X-ray micro computed tomography (CT) were purchased from Sigma–Aldrich Korea (Yongin, Korea). The sectioned mangrove roots were fixed in 2% glutaraldehyde and 2% formaldehyde. Staining was performed by placing the fixed samples in 1% osmium tetroxide for 24 h (osmium tetroxide penetrates into tissues by diffusion). The samples were removed and washed with a sodium cacodylate buffer (0.1 M, pH 7.4) solution and then post-fixed with 0.5% uranyl acetate for 24 h. Specimens were sequentially dehydrated using 30%, 50%, 70%, 90%, and 100% ethanol for 1 h. Then, the specimens were infiltrated using 0%, 25%, 50%, and 75% Spurr’s resin in propylene oxide for 2 h. After overnight infiltration with 100% Spurr’s resin at room temperature, each sample was embedded in Spurr’s resin with the use of a tube and then incubated at 60 °C for 24 h to rigidify the resin.

### X-ray micro CT

The 3D morphological structures of the test samples were observed using X-ray micro CT at the 6C beamline of the Pohang Accelerator Laboratory (PAL, Pohang, Korea). A mechanical shutter was employed to block the X-ray beam except at the instant of image acquisition to minimize photo-thermal damage to the plant sample. The distance from the sample to the camera was determined to be 30 cm to enhance the phase contrast imaging effect. X-ray images were recorded using a charge-coupled device (CCD) camera with a 4008 × 2672 pixel resolution (Vieworks VM-11M, EPIX, USA) and a 1.7 mm × 1.4 mm field of view (FOV). The spatial resolution based on the pixel size of the camera attached with a ×10 objective lens was approximately 10.65 μm/pixel. The sample was firmly fixed to a sample holder that is attached to a rotating stage. The stage was rotated from 0° to 180° at 0.5° intervals. Each acquired image was reduced to 2004 pixels × 1336 pixels using ×2 binning with Octopus software (inCT, Belgium) for rapid data processing. Erroneous spots in the captured X-ray images were removed, and tomograms were reconstructed using Octopus software. The reconstructed images were then analyzed by using Amira software (Visualization Science Group, USA). We employed the modified Bronnikov algorithm filter (inCT, Belgium) in this study to reconstruct with better spatial resolution.

### Sample preparation for SEM

The roots were fixed in 2% glutaraldehyde and 2% formaldehyde, post-fixed in 1% osmium tetroxide, and then dehydrated in a graded ethanol series of up to 100%. Then, the samples were rinsed with 100% ethanol and dried with nitrogen gas to observe their morphological structures. For SEM imaging, the samples were mounted on metal stubs and then coated with platinum (SC7640 model, Quorum Technology, UK). SEM images were obtained by using SEM (JEOL JSM-7401 F, Japan) at an acceleration voltage of 15 kV.

### Pore size measurement

The pore size of the outermost root layer was measured through mercury porosimetry (PoreMaster-60, Quantachrome Co.). The instrument can be used for a wide range of pressures: low pressure (4.83–344.7 kPa) and high pressure (0.12–414 MPa). In this instrument, the low-pressure range was established using high-pressure air, whereas the high-pressure range was created with a hydraulic press by supplying hydraulic oil over the mercury in the dilatometer. In the high-pressure range, the instrument can be used to measure the size of extremely small pores. A test sample was placed in the dilatometer, and then the absorbed and/or adsorbed gases were cleansed by degassing under vacuum conditions (4.83 kPa). Still under vacuum conditions, the dilatometer was filled with mercury. The pressure in the dilatometer gradually increased to the final pressure in the high-pressure and low-pressure tests within approximately 15 min.

### Sodium tetra as a sodium indicator

Sodium tetra (tetramethylammonium) (STTMA, C_84_H_100_C_l4_N_8_O_19_, MW = 1667.5682) was synthesized as previously described^31^,^32^,^33^. The chemical structure of STTMA is depicted in Fig. S2. The synthesized STTMA, which acts as a sodium indicator, was stored at 4 °C or −20 °C to protect from light degradation. Prior to use, the stored STTMA was warmed to room temperature and then suspended with a 20% (w/v) dimethyl sulfoxide (Sigma–Aldrich) solution to render the compound susceptible to hydrolysis. An aqueous solution of STTMA was diluted and freshly used for cell loading. STTMA, one of the tetramethylammonium (TMA) chemicals, is a relatively small molecule with low molecular weight. It can penetrate into the outer and inner layers. These TMA chemicals, such as tetraethylammonium (TEA) and tetrabutylammonium (TBA), have been used to monitor sodium cations in living organisms^34^,^35^,^36^.

Figure S3 shows the typical fluorescence spectra of the excitation and emission of STTMA. The fluorescence spectra were obtained using a Synergy H1 multi-mode spectroscopy analyzer (BioTek, Winoosk, VT). A xenon excitation source with 1 nm intervals was used for both excitation and emission wavelengths. The excitation wavelength for the peak fluorescence intensity was 507 nm, and the emission wavelength was 532 nm, respectively.

### Visualization of sodium ions in the root using two-photon microscopy

A 3.5 wt.% sodium solution was watered into a pot containing the test samples for 20 weeks to visualize the spatial distribution of Na^+^ ions in the outermost root layer. The root was sectioned transversely by using a microslicer (DTK-1000; Dosaka EM, Kyoto, Japan). The thickness of the sectioned slices was approximately 80 μm. The intracellular Na^+^ specific fluorescent indicator STTMA was used to visualize the localization of intracellular free Na^+^. The sectioned slices were incubated in 20 mM MES (2-[N-Morpholino] ethanesulfonic acid) –KOH (pH 6.5) containing 0.5 M mannitol. The sodium-treated roots were soaked in a fluorescent dye solution at 22 ± 3 °C for 12 h (Fig. S4).

The incubated slices were observed under a two-photon laser scanning microscope (Leica Microsystems Ltd. TCS SP5 II MP with SMD, Germany) with a 20× objective lens. The laser power was 1.9 kW (920 nm), and the total exposure time was 230 seconds. The FOV was 775 μm × 775 μm × 90 μm. Morphological structures were consecutively captured at 1 μm depth intervals. The acquired images were processed using LAS AF 2.7 software (Leica Microsystems Ltd. Germany). Outlier noises were removed by using Image J software (National Institutes of Health, USA) to improve image quality.

### Feasibility test under *in vitro* conditions

Basing from the experimental results obtained from mangrove roots, we prepared three types of samples for *in vitro* experiments: normal roots without any pretreatment, roots treated with sodium solution for 4 weeks, and roots treated with HgCl_2_ to block aquaporin. The outermost layer of the samples was considered as a membrane to compare their desalination rates. The experiment was conducted in a centimeter-scale square channel composed of polydimethylsiloxane (PDMS) (Sylgard 184, Dow Corning, USA). The square channel was cured at 80 °C for 3 h. Each of the three membranes was carefully cut and attached at the middle section of the PDMS channel (Fig. 4a). In this experiment model, the outer side of the sliced outermost layer head to upper direction and the inner side face bottom direction. The outer side of the root contact the sodium solution directly like actual root (Fig. 4b). Negative pressure was created inside the channel by sucking at a flow rate of 2 mL/m using a syringe pump (Harvard Apparatus, USA). The syringe pump was activated for 10 min and maintained for 20 min. The negative pressure was measured using a gas pressure sensor (Veaverton, OR, USA). The pressure measurement was conducted for 600 sec while applying negative pressure using a syringe pump. The pressure sensor was connected at the lower part of the PDMS channel, as shown in Fig. S6a. The same experiment was repeated for 10 times to acquire statistically averaged data.

## Additional Information

**How to cite this article**: Kim, K. *et al*. Novel water filtration of saline water in the outermost layer of mangrove roots. *Sci. Rep*. **6**, 20426; doi: 10.1038/srep20426 (2016).

## Supplementary Material

Supplementary Information

## Figures and Tables

**Table 1:** Initial and final conditions of conductivity and pH in the pot.

	Initial conditions (First day)		Final conditions			
	Conductivity (μS/cm)	pH	Max. conductivity (μS/cm)	Final conductivity (μS/cm)	Max. pH	Final pH
1^st^ sodium-treated	1225	7.4	6220	5701	7.7	7.3
2^nd^ sodium- treated	4683	7.2	6435	5315	7.5	7.3
