# Supplementary material for: Novel water filtration of saline water in the outermost layer of mangrove roots
Source: Sci Rep. 2016 Feb 5;6:20426. doi: 10.1038/srep20426 (PMC4742776; doi:10.1038/srep20426)
Supplement: Supplementary Information [file srep20426-s1.doc]

**Novel water filtration of saline water in the outermost layer of mangrove roots**

Kiwoong Kim^1^, Eunseok Seo^2^, Suk-Kyu Chang^3^, Tae Jung Park^3^, and Sang Joon Lee^*, 1^

^1^Department of Mechanical Engineering, Pohang University of Science and Technology (POSTECH), San 31, Hyoja-dong, Pohang 790-784, Republic of Korea

^2^Department of New Biology, Daegu Gyeongbuk Institute of Science and Technology

(DGIST), 333, Techno Jungang-daero, Daegu, 711-873, Republic of Korea

^3^Department of Chemistry, Chung-Ang University, 84 Heukseok-ro, Dongjak-gu,

Seoul 06974, Republic of Korea

^*^Correspondence

Tel: +82-54-279-2169, Fax: +82-54-279- 3199, E-mail: [sjlee@postech.ac.kr](mailto:sjlee@postech.ac.kr)

**Supplementary information**

**
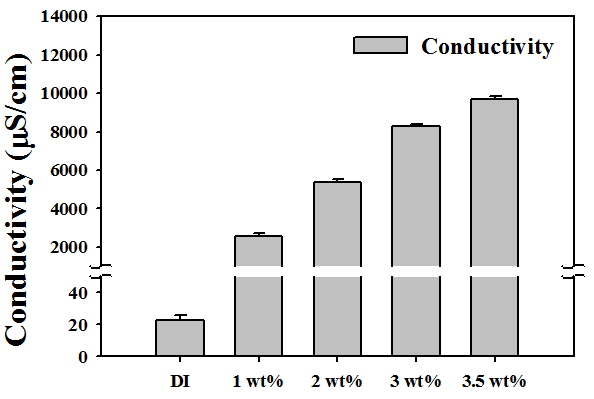
**

**Fig. S1. Variation in conductivity according to sodium solution concentration.**

**
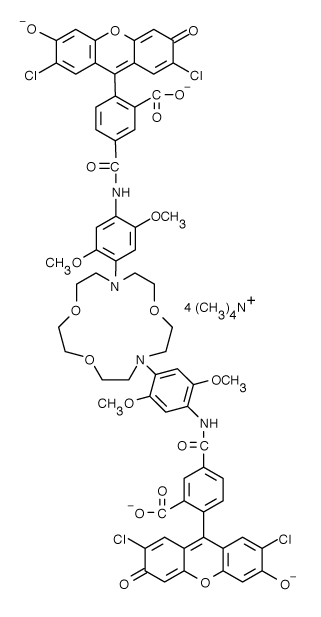
**

**Fig. S2. Chemical structure of STTMA.**

**
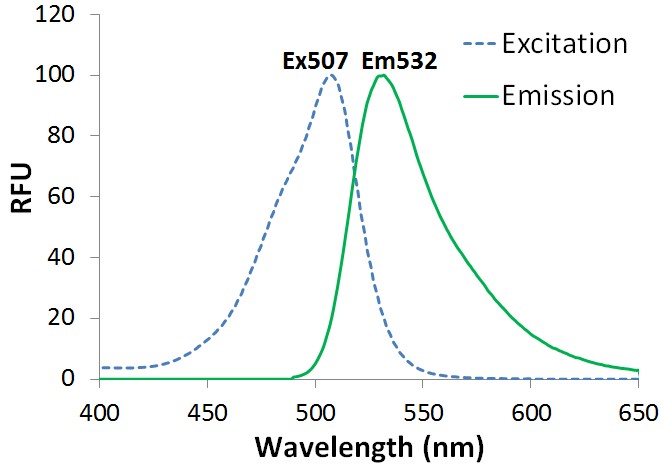
**

**Fig. S3. Fluorescence spectra of STTMA as a sodium indicator.**

**
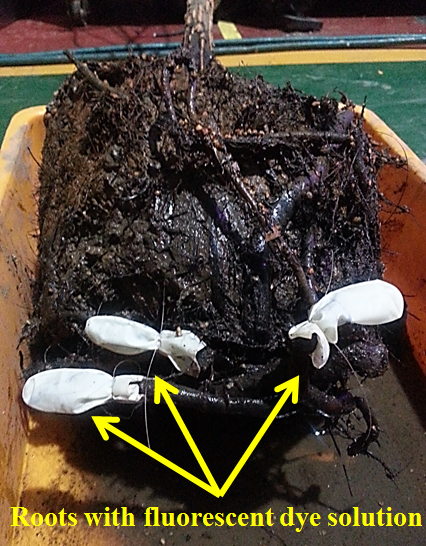
**

**Fig. S4. Soaking of sodium-treated roots in fluorescent dye solution.**


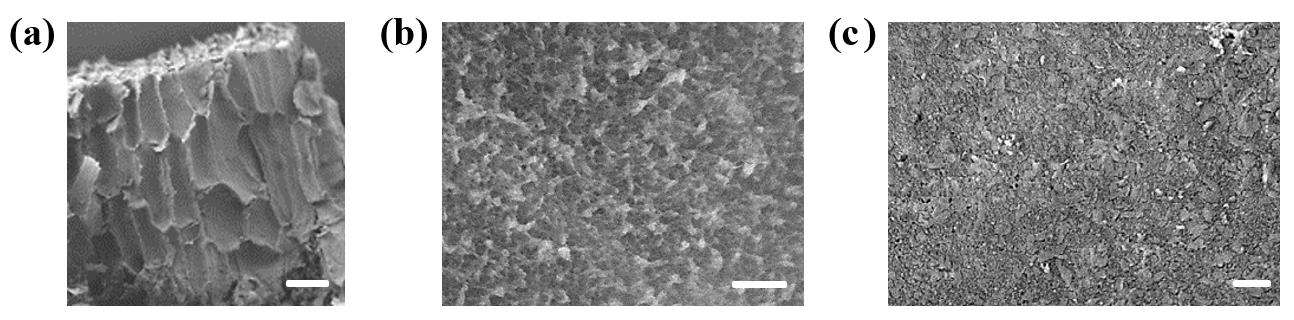


**Fig. S5. SEM images of the outermost layer of RS roots.** (a) Structure of the first layer. (b) Magnified image of (a). (c) Surface pattern of the root. Scale bars in (a), (b), and (c) indicate 25 μm, 1 μm, and 500 nm, respectively.


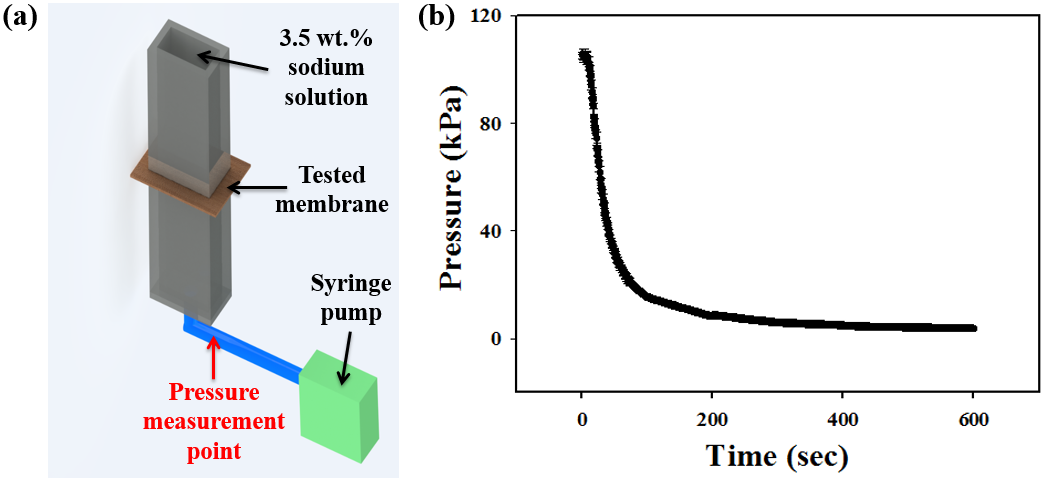


**Fig. S6. Variation of negative pressure applied at the test membrane.** (a) Schematic diagram of experiment setup. (b) Variation of hydrostatic pressure at the test membrane while applying negative pressure with a syringe pump.
